# Health governance in Sub-Saharan Africa

**DOI:** 10.1177/1468018115600123d

**Published:** 2015-12

**Authors:** Keneilwe Sadie Mooketsane, Molefe B Phirinyane

**Affiliations:** University of Botswana, Botswana; Botswana Institute for Development Policy Analysis, Botswana

The advent of global health governance means health administration that transcends national boundaries. In view of the interdependence of states and increasing movement of people, the spread of contagious diseases and the heightened complexity of global health issues make cooperation among countries to be indispensable. Unfortunately resourcing remains a critical challenge to effective health governance. The uncontrollable spread of Ebola in West Africa in 2014 is a good case example of the impact of limited financial and human resources in combating highly contagious diseases that are a potential global threat to human life. It is important to note from the onset that health governance borders on human rights, essentially the right to life. It is for this reason that health needs to remain a top priority for all nations, rich and poor. The disparity in terms of the quality of healthcare and health service provision in developed and developing countries is apparent.

‘Good governance in health systems promotes effective delivery of health services’ (Lewis and Petterson, 2009: 2). Sub-Saharan Africa’s performance on health outcomes remains poor despite Sub-Saharan African nations’ financial commitment, albeit limited, and foreign aid. There is widespread poor service delivery, poor procurement systems marred by mismanagement and corruption, and poor health infrastructure. Many indicators for health outcomes show slow progress in Sub-Saharan Africa. Infant mortality rates per 1000 live births, for example, dropped slightly between 1990 and 2013 from 107 to 66 while comparatively for the Euro area they remained at a single digit (from 8 to 3) in the same period (The World Bank, 2015). There is also the challenge of inadequate resources, weak health systems in terms of access, quality, weak human and institutional capacities. These negative situations are exacerbated by brain drain to richer countries, natural calamities and manmade disasters, especially war. Poor funding by governments is a major contributing factor as the effectiveness of interventions on health is related to the availability of resources. In 2010, Sub-Saharan Africa spent 6.5% of the gross domestic product (GDP) on health which was below the world average of 10.5% of GDP (The World Bank, 2012: 102).

Africa is faced by several other challenges such as conflict, poverty, unemployment, food security, climate change, inequality, industrialisation, among others. This makes prioritising health difficult due to competing demands. As a result, health outcomes tend to correlate with donor support. While international organisations should ideally be driven by member states’ interest, the determination of health priorities by international organisations leads to an imbalance between the set priorities and prevalent health issues in various countries. Sub-Saharan Africa was the only region in the world with external resources incurred on health expenditure that ran in double digits. In South Asia, external resources amounted to only 2.3% of the 2010 health expenditure while for Sub-Saharan Africa it stood at 10.5% of health expenditure (The World Bank, 2012). The rest of the regions in the world received less than 1% of external resources for their health expenditure.

The key question is *what is the role of national actors in global health governance?* One may wonder whether the presence of cooperation and international organisations addressing health matters at a global level does not breed heavy reliance on such organisations and in turn result in diminished capacities for developing nation states. Second, one might ask if the assistance provided through global governance is sufficient. It is indisputable that epidemics such as HIV/AIDS, Swine Flu and Ebola could only be addressed through joint effort with the global community. However, it is also true that governments are the first stop, that is, it is the responsibility of the government of a state nation to provide health services to its people. Therefore, it is important that countries be capacitated to at least manage situations while awaiting aid from international organisations. Regional organisations such as the African Union (AU) and its related sub-regional bodies such as the SADC, Common Market for Eastern and Southern Africa (COMESA) and Economic Community for West African States (ECOWAS) should ensure that member states are capacitated to handle disease epidemics and other natural disasters such as floods that can spread contagious diseases. The coordination of this cooperation needs to be strengthened and availed the necessary resources and mandates to be able to effectively coordinate the control of communicable and NCDs.

The AU and its sub-regions should establish early warning systems about epidemics, natural disasters and other threats that may derail health governance in the region. Such an arrangement calls for closer coordination of national health policies in the sub-regions and in particular the ratification and domestication of health protocols. A healthy worker is a more productive worker, and if Sub-Saharan Africa leaders want to sustain the economic growth of their countries, then they have to increase health expenditure.

While it is important for individual countries to adopt measures specific to their circumstances, the AU and sub-regional bodies should adopt a new approach to dealing with donors. Instead of donors only strengthening bilateral relations with recipient countries, donor funding should be channelled through regional bodies to address challenges of a regional nature such as combating the HIV/AIDS pandemic, Ebola, Malaria and so on. The AU’s decision in 2013 to establish the African Centre for Disease Control (ACDC) is a good initiative. However, a firm commitment to ACDC by the AU came only after the Ebola epidemic hit West Africa and notably after the US Centres for Disease Control and Prevention promised assistance in the form of technical expertise and advice. This development shows Africa’s heavy reliance on foreign aid for initiatives that are of a strategic nature as this one.

Financial resources are not really a major challenge for Sub-Saharan Africa as it is usually perceived. According to the International Monetary Fund (IMF), Sub-Saharan Africa’s economic growth remained robust at 5.2% in 2014, having been at 6.3% in 2013. The IMF further indicates that capital inflows to Sub-Saharan Africa made 5.3% of regional GDP in 2013, which was higher than the developing countries’ average of 3.9%. Revenue from tourism also made significant financial contribution to the regions’ balance sheet. Many countries in Africa spend heavily on arms due to internal conflicts (see Horn Affairs (2011)^1^). Of course, there are numerous threats to Sub-Saharan Africa’s growth prospects, but notwithstanding these threats the region seems poised for better prospects. It would seem that the best strategy to consolidate the prospect of long-term sustained economic growth is through deepened regional cooperation and entrenching good governance. The impact of Ebola on the economies of some West African countries is a present reminder of what disease outbreaks can do to economic growth. Health governance should be given the appropriate significance that it deserves if growth rates are to be sustained.

Furthermore, developing countries need to develop strategies of collaboration between governments and non-state actors. The question that arises is ‘are Sub-Saharan Africa countries adequately tapping on alternative sources of assistance?’ Many Sub-Saharan Africa countries still view non-state actors with suspicion. But those that have embraced them as development partners have reaped some positive results in the provision of health services. In Botswana, for example, four of the eight hospitals that operate within 100 km of Gaborone, where half the population of the country lives, are mission hospitals while the private and public sectors own two each. The role played by the church in Malawi’s health sector is one of the positive examples of this collaboration. In Malawi, for instance, the government pays the salaries of the mission hospitals (Rookes and Rookes, 2012). The hospitals enjoy some degree of autonomy to the extent they are able to manipulate their governance systems to suit the situation they operate in Rookes and Rookes (2012).

A multipronged strategy of cooperation between the African states, regional bodies, religious organisations or civil society, the private sector and donors could most likely be the winning strategy and should be enhanced and encouraged by both the AU and influential donors. The porous borders in Africa and limited resources in terms of health services provision necessitate cooperation across national boundaries to effectively combat the spread of diseases.

## Notes

1. http://hornaffairs.com/en/2011/06/12/africa-top-25-military-spending-countries/

## References

[bibr1-1468018115600123] Horn Affairs (2011) Africa: Top 25 Military Spending Countries. Available at: http://hornaffairs.com/

[bibr2-1468018115600123] Lewis M Petterson G (2009) Governance in health care delivery: Raising performance. Policy Research Working Paper no. 5074. Washington, DC: The World Bank.

[bibr3-1468018115600123] Rookes P Rookes J (2012) Commitment, Conscience or Compromise: The Changing Financial Basis and Evolving Role of Christian Health Services in Developing Countries. Saarbrücken: LAP Lambert Academic Publishing GmbH & Co. KG.

[bibr4-1468018115600123] The World Bank (2012) World Development Indicators 2012. Washington, DC: The World Bank.

[bibr5-1468018115600123] The World Bank (2015) World Development Indicators 2015. Washington, DC: The World Bank Available at: http://elibrary.worldbank.org

